# Clinical Profile and Long-Term Outcomes of Scrub Typhus Meningoencephalitis in Children

**DOI:** 10.7759/cureus.79055

**Published:** 2025-02-15

**Authors:** Hricha Mishra, Chandra Kanta, Amita Jain, Ravindra Kumar Garg, Shantanu Prakash, Rajkumar Kalyan

**Affiliations:** 1 Pediatrics, King George's Medical University, Lucknow, IND; 2 Microbiology, King George's Medical University, Lucknow, IND; 3 Neurology, King George's Medical University, Lucknow, IND

**Keywords:** acute encephalitis syndrome (aes), children, clinical presentation, meningoencephalitis, outcome, scrub typhus

## Abstract

Introduction: Scrub typhus is a significant cause of acute encephalitis syndrome (AES) in pediatric populations, particularly in northern India. Its long-term outcomes have not been well studied to date. This study investigates the clinical profile and long-term outcomes of scrub typhus meningoencephalitis in children.

Methods: This prospective observational study was conducted at King George’s Medical University, Uttar Pradesh, India, from August 2018 to October 2020. Children aged 0.3 months to 14 years who presented with AES were tested for scrub typhus using serum IgM Enzyme-Linked Immunosorbent Assay (ELISA) and polymerase chain reaction (PCR) of cerebrospinal fluid (CSF) and peripheral blood mononuclear cells (PBMC). Positive patients were followed up for one year to assess long-term neurological outcomes.

Results: Of 264 children tested for scrub typhus, 78 (29.5%) were positive. Neurological symptoms included altered sensorium (70 (89.74%)), seizures (69 (88.46%)), and focal neurological deficits 8 (10.26%). Hepato-splenomegaly (27 (34.61%)), thrombocytopenia (48 (61.5%)), and raised liver transaminases (51 (65.4%)) were the other common manifestations. Mortality occurred in 10.25% of cases. Sixty-seven cases were followed up for one year. Long-term outcomes indicated that 60 (89.55%) of the patients fully recovered, while seven (10.45%) experienced sequelae, including minor impairment in four (5.97%), moderate impairment in two (2.99%), and severe impairment in one case (1.49%).

Conclusion: Scrub typhus is a major cause of AES in children, with significant neurological sequelae in a small number of cases.

## Introduction

Scrub typhus, caused by the bacterium* Orientia tsutsugamushi*, is difficult to diagnose due to the overlap of non-specific symptoms with other conditions and a lack of evidence on the effectiveness of diagnostic testing [1]. Patients show non-specific signs and symptoms, such as fever, headache, myalgia, coughing, abdominal pain, and diarrhea, which cannot be distinguished from those of other systemic illnesses; this is a defining trait of the illness. Eschar, which is only present in some patients, may help in making a clinical diagnosis of this condition [2]. Scrub typhus usually presents with moderate clinical symptoms and responds well to antibiotic therapy; nevertheless, in situations of late diagnosis, significant complications can emerge, potentially leading to multi-organ failure and death [2]. Organisms disseminate from the skin to target organs, and *O. tsutsugamushi* can be demonstrated in peripheral white blood cells taken from patients presenting to the hospital with acute scrub typhus [3]. Rural parts of Southeast Asia, such as India, Thailand, Korea, Australia, Russia, the Pacific Islands, and Japan, have a higher incidence of this illness. According to passive national monitoring systems, the seroprevalence of this disease ranges from 9.3% to 27.9%, with a death rate of up to 30%, or possibly higher, among untreated individuals [4,5]. Although the disease is common in India, it remains significantly underdiagnosed due to its non-specific clinical presentation, limited access to specialized diagnostic facilities in many areas, and low clinical suspicion among healthcare providers [5].

In recent years, various studies have documented that scrub typhus is the most common cause of acute encephalitis syndrome (AES) in Uttar Pradesh, India [6]. The pediatric population is more affected than adults [6]. Few studies describe the clinical presentation of scrub typhus meningoencephalitis, but there is a lack of research on its long-term outcomes in children. The patient's neurological aftereffects can limit their ability to engage and integrate into society.

In the current study, we followed the patients for one-year duration to find out if there were any complications from scrub typhus encephalitis. We also aimed to characterize the clinical presentation and laboratory tests in this study to better understand the clinical features and the specific investigations required to diagnose this illness. This will help develop a framework that supports prompt intervention and therapy, enabling early decision-making. Additionally, it will facilitate improved decision-making to enhance patients' prognosis and quality of life.

So, this study was done to observe the clinical profile and outcomes of scrub typhus meningoencephalitis in children at one-year follow-up.

This article was previously presented as a poster at the SAPA-SLCP International Pediatric Conference on June 10th, 2024.

## Materials and methods

Study setting

This study was conducted in the Department of Pediatrics at King George's Medical University, Lucknow, India, over a period of two years and three months, from August 2018 to October 2020. The university is a public tertiary care teaching hospital that primarily admits seriously ill and underserved patients from the city and surrounding districts. Laboratory investigations were conducted in the Department of Microbiology and the Department of Pathology at the university. Cases were enrolled from August 2018 to October 2019 and followed up for one year.

Ethics statement

The study was approved by the Institutional Ethics Committee (reference code: 89thECM II B-PhD/P3/2018). Patients were enrolled after obtaining written informed consent from the parents and ascent from patients (when applicable). 

Enrolment of cases and data collection

The study included children aged three months to 14 years who presented with an acute onset of fever ≥38°C (100.4°F) and altered mental status lasting ≥24 hours and/or new-onset seizures not fully attributable to a pre-existing seizure disorder. The case definition of AES was set according to the WHO protocol [7]. After receiving written informed consent from the parents, blood samples and CSF samples (when there was no contraindication for doing a lumbar puncture) were withdrawn. Patients of CNS tuberculosis, epilepsy, trauma, toxin exposure, cerebrovascular accidents, and malignancy were excluded. Clinical and laboratory details of scrub typhus-positive cases were recorded on a predesigned proforma. 

Laboratory investigations

All cases were tested for serum IgM antibodies against scrub typhus using Enzyme-Linked Immunosorbent Assay (ELISA). Peripheral blood mononuclear cells (PBMC) and cerebrospinal fluid (CSF) were tested by polymerase chain reaction (PCR) for scrub typhus. Those who tested positive by any of the following methods (ELISA, CSF PCR, or PBMC PCR) were considered positive for scrub typhus.

Anti-scrub typhus IgM antibodies were evaluated in serum samples at the Microbiology Department of King George's Medical University, Lucknow, India, using Inbios International, USA kits. Samples with an optical density (OD) >0.5 were considered positive for anti-scrub typhus IgM. Baseline titers for scrub typhus IgM must be determined for each location; for India, this value has been established as 0.5 [8]. For CSF and PBMC samples, PCR and DNA extraction were performed using the QI Amp DNA Blood Micro Kit (Qiagen, GmbH, Hilden, Germany). Real-time PCR was conducted by targeting the 47-kDa protein gene.

We also tested for other common etiologies of AES in our region. Anti-dengue virus (DV) IgM and anti-chikungunya virus (Chik V) IgM in serum were tested using kits from the National Institute of Virology, Pune, India. Cut-off values were calculated based on the manufacturer’s instructions. All ELISAs were performed using serum samples, except for the anti-JEV IgM ELISA, which was preferably performed using CSF samples (as per the manufacturer’s recommendations). CSF samples were tested by PCR for herpes simplex virus-1 and scrub typhus [6,9]. Scrub typhus PCR was also performed on PBMC samples. In addition, blood culture, sensitivity testing, and a rapid antigen test for malaria were conducted.

Other tests performed were complete blood counts, serum electrolytes, blood sugar levels, renal function tests, and liver function tests.

Follow-up

All patients positive for scrub typhus were followed up daily during hospitalization and monthly after discharge for one year. They were assessed for long-term outcomes in terms of epilepsy, focal neurological deficits, behavioral problems, intellectual disability, and motor disability.

Those who failed to visit the hospital were contacted telephonically by asking for a set of questionnaires to understand sequelae and mortality in the positive cases. The questionnaire was prepared according to the Liverpool outcome scale (LOS) [10]. This established test was created to evaluate impairment among children following JEV-caused encephalitis and has been proven in India. The assessment evaluates fundamental motor and self-care abilities, along with basic cognitive and behavioral functions, by asking caretakers 10 questions and making five observations of the child engaging in simple tasks.

The scoring system for each question covered a range of values, with five representing complete recovery; four representing minor sequelae that did not impact physical function, personality change, or medication usage; three representing moderate sequelae that mildly affected function and were likely compatible with independent living; two representing severe sequelae that significantly impaired function to the extent of requiring the patient to be dependent; and finally one representing death. The lowest result was obtained for any of the 15 questions/activities, resulting in the outcome score.

Statistical analysis

Data was entered and coded in a Microsoft Excel (Microsoft Corp., Redmond, WA) spreadsheet. Analysis of data was performed using Statistical Package for the Social Sciences (SPSS) software, Version 20.0 (IBM Corp., Armonk, NY, USA). Continuous parametric variables were expressed as means and standard deviation, while continuous non-parametric variables were expressed as median and interquartile range. Categorical variables were expressed as percentages.

Post hoc power analysis was done based on a recently published study [11], in which sequelae were reported in 50% of cases of AES due to scrub typhus. We followed up on 67 cases of scrub meningoencephalitis for one year and found sequelae in only 10% of the cases. Taking an alpha error of 0.05, the study power was calculated to be 100%.

## Results

A total of 264 children presenting with AES were tested for scrub typhus and other etiologies. Of these, 78 (29.5%) cases were positive for scrub typhus: serum IgM antibodies in 61 (23.1%), CSF PCR in 21 (7.9%), and whole blood PBMC in 26 (9.8%) cases, respectively. Forty (15%) cases were positive for dengue IgM, chikungunya IgM, and Japanese Encephalitis (JE) IgM. The etiology remained undiagnosed in 146 (55%) patients. None of the CSF samples tested positive for herpes simplex virus-1 by PCR.

The mean duration of illness before admission to the study hospital was 7.78 days. History of fever and change in mental status were present in all cases. Seventy (89.74%) had altered sensorium at presentation. Sixty-nine (88%) cases had seizures, with generalized tonic-clonic seizures being the most common type, present in 57 (82.6%) cases. The median Glasgow Coma Scale at the time of admission was eight (ranging from 3 to 14). Meningeal signs were positive in 20 (25%) children, and papilledema was present in five (6.41%). Other neurological findings included abnormal movements in six (7.69%), increased tone in 30 (38.46%), focal neurological deficits in eight (10.26%), and signs of raised intracranial pressure in 24 (30.77%). An increased respiratory rate was found in 36 (46.15%), hepatomegaly in 27 (34.61%), and splenomegaly in 20 (25.64%). Rashes and edema were present in 19 (24.36%) and 14 (17.95%), respectively. Eight patients (10.25%) died due to various complications (Table 1).

**Table 1:** Clinical features of enrolled cases (N=78) GCS, Glasgow Coma Scale; IQR, interquartile range; ICP, intracranial pressure

Clinical feature	No (%)
Mean age (years) ± SD	6.86±3.44 (0.3-14)
Male:female ratio	1.11:1
Fever	78 (100%)
Seizures	69 (88.46%)
GCS at presentation (median (IQR))	8.00 (4.00-10.00)
Headache	10 (12.82%)
Vomiting	25 (32.05%)
Diarrhea	7 (8.97%)
Respiratory symptoms	36 (46.15%)
Meningeal signs	20 (25.64%)
Abnormal tone	30 (38.46%)
Papilledema	5 (6.41%)
Focal neurological deficits	8 (10.26%)
Signs of raised ICP	24 (30.77%)
Cranial nerve involvement	3 (3.85%)
Brisk reflexes	40 (51.28%)
Extensor planter	34 (43.59%)
Abnormal movements	6 (7.69%)
Bleeding manifestations	6 (7.69%)
Shock	15 (19.23%)
Edema	14 (17.95%)
Hepatomegaly	27 (34.61%)
Splenomegaly	20 (25.64%)
Lymphadenopathy	4 (5.12%)
Eschar	3 (3.85%)
Rashes	19 (24.36%)
Mortality	8 (10.25%)

Table 2 shows the laboratory findings of enrolled cases. The mean total leukocyte count was found to be elevated. Thrombocytopenia (platelet count <1.5 lac/µL) was found in 48 (61.5%) cases, and raised liver transaminases were observed in 51 (65.4%) cases. CSF examination revealed mild pleocytosis and a mild increase in protein levels.

**Table 2:** Laboratory findings of enrolled cases (N=78) TLC, total leukocyte count; AST, aspartate aminotransferase; ALT, alanine aminotransferase; Na^+^, sodium; K^+^, potassium; CSF, cerebrospinal fluid

Laboratory parameter		Value (±SD)
Hemoglobin (g/dL)		9.48 (±1.53)
TLC (per cu mm)		18,508.41 (±15,351.51)
Neutrophils (%)		60.79 (±14.06)
Lymphocytes (%)		35.41 (±13.61)
Platelet count (lac/cu mm)		1.3 (±0.72)
Serum bilirubin (mg/dL)		0.77 (±0.82)
AST (IU/L)		114.59 (±95.27)
ALT (IU/L)		90.73 (±68.6)
Serum Na+ (mmol/L)		138.4 (±5.25)
Serum K+ (mmol/L)		4.45 (±0.59)
Serum creatinine (mg/dL)		0.75 (±0.48)
Blood urea (mg/dL)		41.95 (±24.36)
CSF findings	CSF cells (cumm)	20.03 (±21.36)
	CSF neutrophil (%)	24.1 (±14.61)
	CSF lymphocytes (%)	75.76 (±14.7)
	CSF glucose (mg/dL)	63.58 (±20.36)
	CSF protein (mg/dL)	144.48 (±70.02)

A total of eight (10.25%) cases of 78 expired. Two of the deceased patients tested positive for dengue and chikungunya along with scrub typhus. Three cases were lost to follow-up. Subsequent data from our study based on LOS indicates that four (5.97%) children had minor sequelae, two (2.99%) had moderate, and one (1.49%) had severe sequelae. The rest of the children (60 (89.55%)) recovered fully. One child developed epilepsy, and one had a persistent focal neurological deficit. Table 3 shows the distribution of cases according to complications experienced after one-year follow-up.

**Table 3:** Outcome of enrolled cases at one-year follow-up

Outcome after one-year follow-up (N=67)		Number of cases	Percentage
Outcome (Liverpool outcome scale)	Full recovery	60	89.55%
	Minor sequelae	4	5.97%
	Moderate sequelae	2	2.99%
	Severe sequelae	1	1.49%
Focal neurological deficit		1	1.49%
Epilepsy		1	1.49%

## Discussion

This prospective observational study was done at a tertiary care teaching hospital in northern India. The thorough approach, methodology, and insightful results of this study significantly advance our knowledge of the clinical profile and long-term consequences of scrub typhus in children. Scrub typhus is a neglected tropical disease and now it is the most common cause of AES in children in Uttar Pradesh. Focusing on children gives valuable insights into the effects of scrub typhus on developing brains. 

This study's findings show that scrub typhus is a major cause of AES in children in Uttar Pradesh, India. Of 118 (44%) patients with a known etiology, a microbiological test for *O. tsutsugamushi *was positive in 78 (66%) out of 118 patients, making it the most common etiology obtained in the study. A growing number of research efforts in Asia have shown *O. tsutsugamushi'*s* *contribution to the continent's burden of acute febrile illness, including South Korea, Japan, China, Taiwan, Thailand, and Bhutan, where scrub typhus is a notifiable disease [12]. *O. tsutsugamushi* was found in 1-4.7% of children in Cambodia, Vietnam, Laos, Myanmar, and Thailand, according to studies that included screening for it as part of comprehensive surveillance of childhood CNS diseases [13,14]. Interstitial pneumonia, acute renal failure, meningoencephalitis, gastrointestinal hemorrhage, and multiple organ failures are among the severe clinical symptoms or complications of scrub typhus that have been reported. Scrub typhus patients may pass away from such consequences; hence we should closely monitor this disease condition [15]. In this study, most of the positive cases (71 (91.03%)) were reported during the month of August to November, and almost all children were from Uttar Pradesh. In this study, the number of males was slightly higher than females, 42 (53%), which may be related to the fact that males tend to play outside in vector-ridden vegetation [16]. All cases had a fever, and 90% of the children experienced it for five to 14 days. Altered sensorium was observed in 70 (89.74%) cases, and seizures were the second most prevalent symptom in this study, which may be higher than what was observed in other studies [17]. Similar to this study [18], vomiting was reported in 25 (32%) cases and headache in 10 (13%). Since dengue, JE, scrub typhus, and many other infections occur in the same months of the year and share symptoms, including fever, altered sensorium, seizures, headache, abdominal discomfort, and rash, diagnosing any one of these can be challenging.

Other than CNS manifestations, a significant proportion of cases had hepato-splenomegaly and respiratory symptoms. As scrub typhus involves multiple systems, these features have also been reported in 55.45% and 47.54% of cases by other authors [19]. Rash and eschar were found in 19 (24.36%) and three (3.85%) cases, respectively, in the present study.

We studied the hematological and biochemical values of scrub typhus-positive cases and found that the leukocyte count was higher in our study, but other studies have reported differently [20]. The mean hemoglobin and platelet counts were lower compared to non-scrub typhus-positive cases [21-23]. Thrombocytopenia was found in 48 (61.5%) cases, and raised liver transaminases were observed in 51 (65.4%) cases. Other studies have also reported that thrombocytopenia (77%) and elevated liver transaminases (64%) are common among cases of scrub typhus [21]. In this study, the mean CSF lymphocyte count was mildly elevated, and CSF protein was also on the higher end, similar to what others have reported [22]. However, a few studies contradict these figures [23]. CSF glucose was not decreased in the majority of cases.

Although the mortality rate varies from state to state and country to country, it can be as high as 70% in untreated cases. However, in our study, the mortality rate of scrub typhus was 8 (10.25%) [12]. Mortality in scrub typhus is not solely due to complications of meningoencephalitis; the involvement of other systems also plays a role. Often, patients experience multiorgan dysfunction during the final stage.

Other studies, though very few, have shown that sequelae can occur in scrub typhus cases if not properly treated. These studies have reported cortical damage involving language and psychomotor components, linguistic delay, peripheral neuropathy, hearing loss, urine, and fecal incontinence, as well as cerebellar, vestibular, and motor dysfunction [24]. Our study also showed sequelae such as neurologic deficits, speech difficulties, and abnormal mental health. Some studies have reported no sequelae at all in their patients, as they initiated proper antibiotic treatment at the appropriate time [25].

Studies on JE survivors revealed that 27% to 50% of them had moderate to severe disabilities. Common outcomes included seizures, urinary incontinence, behavioral changes, the need for assistance with dressing, the inability to be alone without risk, and a decline in school or work performance. Compared to JE, the proportion of survivors of AES scrub typhus with moderate to severe impairments was lower [26].

In this study, we noticed complications in seven (10%) patients after discharge from the hospital compared to other studies [27]. Another study from Uttar Pradesh has also documented that sequelae in AES are more common in JE-positive cases compared to non-JE cases in children [28].

A recently published study [11] reported sequelae in 50% of AES cases due to scrub typhus. In contrast, the rate of sequelae was significantly lower in our study. Differences in sequelae may be attributed to variations in the severity of cases admitted and the timing of treatment initiation for scrub typhus. As per departmental protocol, empirical treatment with doxycycline or azithromycin for scrub typhus is started at the time of admission for any child with AES, before the confirmation of a definite etiological diagnosis.

The suspicion of scrub typhus and prompt, proper treatment are crucial in preventing deaths and needless sequelae from the condition.

Limitation of the study and future prospect

The available data for follow-up of scrub typhus do not allow us to make generalizable conclusions about sequelae, as the study was conducted at a single center and focused solely on a specific group. Therefore, the results of sequelae may not be applicable to other populations from different geographic areas.

Some participants were unable to attend follow-up visits in person due to financial constraints, living far from our site, and later due to COVID-19. As a result, these cases were followed up telephonically by conversing with their parents about their health.

## Conclusions

In conclusion, our study reveals that scrub typhus is a common cause of AES in children in Lucknow and the surrounding districts of Uttar Pradesh, India. These AES cases often present with multisystem involvement, and hematological, respiratory, cardiac, and hepatic involvement is common. Since it is often difficult to differentiate AES due to scrub typhus from other causes of AES at presentation, empirical antibiotic therapy should be considered for all AES cases to improve outcomes in endemic areas.

These children should be followed up regularly to identify disabilities early and referred to rehabilitative services, although we found that only a small percentage developed long-term complications.
